# Supplementary material for: Bibliometric Analysis and a Call for Increased Rigor in Citing Scientific Literature: Folic Acid Fortification and Neural Tube Defect Risk as an Example
Source: Nutrients. 2024 Aug 1;16(15):2503. doi: 10.3390/nu16152503 (PMC11313885; doi:10.3390/nu16152503)
Supplement: Supplementary file 1 [file nutrients-16-02503-s001.zip › nutrients-3097562-supplementary.pdf]

nutrients

MDPI

Supplementary

Bibliometric Analysis and a Call for Increased Rigor in Citing Scientific Literature: Folic Acid Fortification and Neural Tube Defect Risk as an Example

Brynne Boeck [1] and Cara J. Westmark [1,2,*]

[1] Department of Neurology, University of Wisconsin, Madison, WI, USA; bboeck@wisc.edu
[2] Molecular Environmental Toxicology Center, University of Wisconsin, Madison, WI, USA; westmark@wisc.edu
* Correspondence: westmark@wisc.edu; Tel.: 1-608-262-9730

Table S1. BIBLIO checklist for reporting bibliometric reviews of the biomedical literature[1]

Section/topic	Item	Checklist	Reported on
**Title**			
Identification	1	Identify the report as a bibliometric review in the title	Line 2
Issues/topics	2	Indicate the key issues/topics under investigation and coverage of time period	Lines 3-4, 93
**Abstract**			
Structured summary	3	Summary including (as applicable): background, methods, results and conclusions	Lines 10-25
**Introduction/background**			
Justification/rationale/explanation	4	Present review of existing knowledge and epidemiological information	Lines 29-41
Objectives	5	Statement of the objective(s) or question(s)	Lines 86-89
**Methods**			
Search engines (data sources)	6	Describe all information sources (such as electronic databases)	Lines 93-94
Search strategy	7	Keywords and systematization criteria (date of search, language, type of document) for the search	Lines 93-95
Time period	8	The period that the review covers	Line 94
Eligibility criteria	9	Describe all inclusion and exclusion criteria, languages, study design, type of publication, and time period	Lines 96-99
Data refinement (selection procedure)	10	Remove the irrelevant articles, inspection to eliminate duplicate and unrelated articles	Lines 96-97
Quality Assessment	11	Assessment of papers by multiple authors and the use of assessing checklists	Lines 106-107
Data synthesis	12	Describe the methods used for summarizing, handling, synthesis, tabulations, or schematic displays. Describe how the data were analyzed	Lines 113-115
**Results**			
Descriptive findings (statistics)	13	Provide details of the search and selection process and the number of citations retrieved	Line 120
Schematic map and trend	14	Summarize and/or present the schematic maps and trends	Lines 124-126
Tabulation & summarizing findings	15	Start the presentation with a historical view (who and when first published on the topic), report on review papers, summarize the findings according to outcome measures or populations	Lines 118-119
Synthesis of findings	16	Synthesize the findings as much as possible, find the gap, and propose a model, hypothesis, etc. (if applicable)	Lines 118-151, Table 1

**Discussion**			
Summary of evidence	17	Summarize the main findings. The findings should be presented in more "general" or "accessible" terms	Lines 205-206, 209-210
Interpretation	18	Include interpretation consistent with results. Explanation for observed outcomes, similarities, and differences reported	Lines 210-219
Strengths and limitations	19	Discuss the strengths and limitations	Lines 340-344
Conclusion	20	Provide a general interpretation of the results with respect to the review questions and objectives, as well as potential implications	Lines 345-365

[1] Adapted from Montazeri, A.; Mohammadi, S.; M Hesari, P.; Ghaemi, M.; Riazi, H.; Sheikhi-Mobarakeh, Z. Preliminary guideline for reporting bibliometric reviews of the biomedical literature (BIBLIO): a minimum requirements. *Syst Rev* **2023**, *12*, 239, doi:10.1186/s13643-023-02410-2.
